# Predictors of lung cancer subtypes and lymph node status in non-small-cell lung cancer: intravoxel incoherent motion parameters and extracellular volume fraction

**DOI:** 10.1186/s13244-024-01758-w

**Published:** 2024-07-06

**Authors:** Huizhi Han, Wenxiu Guo, Hong Ren, Huiting Hao, Xiangtao Lin, Mimi Tian, Jiaxiang Xin, Peng Zhao

**Affiliations:** 1 Department of Radiology, Shandong Provincial Hospital Affiliated to Shandong First Medical University, Jinan, China; 2 MR Research Collaboration, Siemens Healthineers Ltd, Shanghai, China

**Keywords:** Extracellular volume fraction, Intravoxel incoherent motion, Lung cancer, Lymph node

## Abstract

**Objective:**

To determine the performance of intravoxel incoherent motion (IVIM) parameters and the extracellular volume fraction (ECV) in distinguishing between different subtypes of lung cancer and predicting lymph node metastasis (LNM) status in patients with non-small-cell lung cancer (NSCLC).

**Methods:**

One hundred sixteen patients with lung cancer were prospectively recruited. IVIM, native, and postcontrast T1 mapping examinations were performed, and the T1 values were measured to calculate the ECV. The differences in IVIM parameters and ECV were compared between NSCLC and small-cell lung cancer (SCLC), adenocarcinoma (Adeno-Ca) and squamous cell carcinoma (SCC), and NSCLC without and with LNM. The assessment of each parameter’s diagnostic performance was based on the area under the receiver operating characteristic curve (AUC).

**Results:**

The apparent diffusion coefficient (ADC), true diffusion coefficient (*D*), and ECV values in SCLC were considerably lower compared with NSCLC (all *p* < 0.001, AUC > 0.887). The *D* value in SCC was substantially lower compared with Adeno-Ca (*p* < 0.001, AUC = 0.735). The perfusion fraction (*f*) and ECV values in LNM patients were markedly higher compared with those without LNM patients (*p* < 0.01, < 0.001, AUC > 0.708).

**Conclusion:**

IVIM parameters and ECV can serve as non-invasive biomarkers for assisting in the pathological classification and LNM status assessment of lung cancer patients.

**Critical relevance statement:**

IVIM parameters and ECV demonstrated remarkable potential in distinguishing pulmonary carcinoma subtypes and predicting LNM status in NSCLC.

**Key Points:**

- Lung cancer is prevalent and differentiating subtype and invasiveness determine the treatment course.
- True diffusion coefficient and ECV showed promise for subtyping and determining lymph node status.
- These parameters could serve as non-invasive biomarkers to help determine personalized treatment strategies.

**Graphical Abstract:**

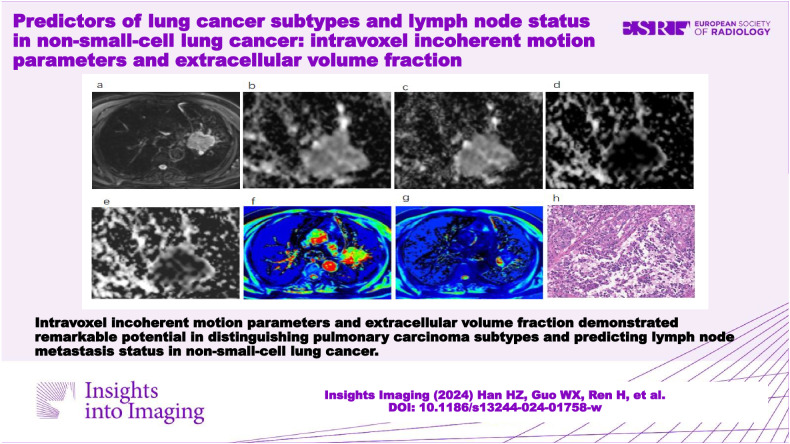

## Introduction

Lung cancer is one of the leading causes of cancer-related deaths globally. Primary lung cancer is predominantly classified into small-cell lung cancer (SCLC) and non-small-cell lung cancer (NSCLC), with NSCLC accounting for approximately 85% of cases. The most prevalent pathologic subtypes of NSCLC are adenocarcinoma (Adeno-Ca) and squamous cell carcinoma (SCC) [1]. SCLC is characterized by high invasiveness and a propensity for early metastasis, with treatment primarily focused on radiation therapy and chemotherapy [2]. In contrast, the treatment for NSCLC is closely related to factors such as the presence of lymph node metastasis (LNM) and clinical staging. Notably, patients without LNM exhibit a considerably improved prognosis than those with lymph node involvement [3]. Therefore, early differentiation of lung cancer histologic types and accurate assessment of LNM status are pivotal for devising personalized treatment plans and improving prognosis in NSCLC.

The occurrence of LNM is related to the elevated interstitial fluid pressure and hypoxia of the primary lesion [4]. Various lung cancer subtypes manifest distinctions in extracellular space and angiogenesis [5]. Based on these principles, magnetic resonance imaging (MRI), capable of indicating perfusion and cellularity information within the tumor microenvironment, is expected to serve as a tool for distinguishing pulmonary carcinoma subtypes and predicting LNM status in NSCLC. Intravoxel incoherent motion (IVIM) provides information on both diffusion and perfusion in tissues [6]. It performs well in discerning between malignant and benign tumors, distinguishing the pathological classification of tumors, and assessing treatment response [6–8]. Several research studies have shown that the true diffusion coefficient (*D*) is a reliable marker reflecting the extracellular space. It is not affected by microcirculation perfusion and exhibits higher performance than the apparent diffusion coefficient (ADC). Meanwhile, the extracellular volume fraction (ECV) is calculated based on T1 values of lesions and arteries before and after contrast agent injection. It signifies the proportion of extracellular space, providing insights into the microvascular density and stromal fibrosis degree [9]. It is increasingly used for evaluating liver fibrosis, tumor grade, and post-chemotherapy response [9–11]. A recent study has demonstrated the capability of ECV in identifying lymphovascular stromal infiltration in patients with cervical cancer [12]. Therefore, IVIM parameters and ECV have the potential to differentiate lung cancer subtypes and predict LNM in NSCLC.

There is limited research on the use of IVIM parameters and ECV derived from primary lesions to predict LNM in NSCLC, and additional validation of the findings is required. Moreover, the variations in IVIM parameters and ECV among different subtypes of lung cancer are largely unexplored. Given these considerations, this study purposed to clarify whether IVIM parameters and ECV could differentiate pulmonary carcinoma subtypes and predict the LNM status of NSCLC while also comparing their diagnostic performance, assisting in clinical decision-making.

## Materials and methods

### Patients

**Fig. 1:**
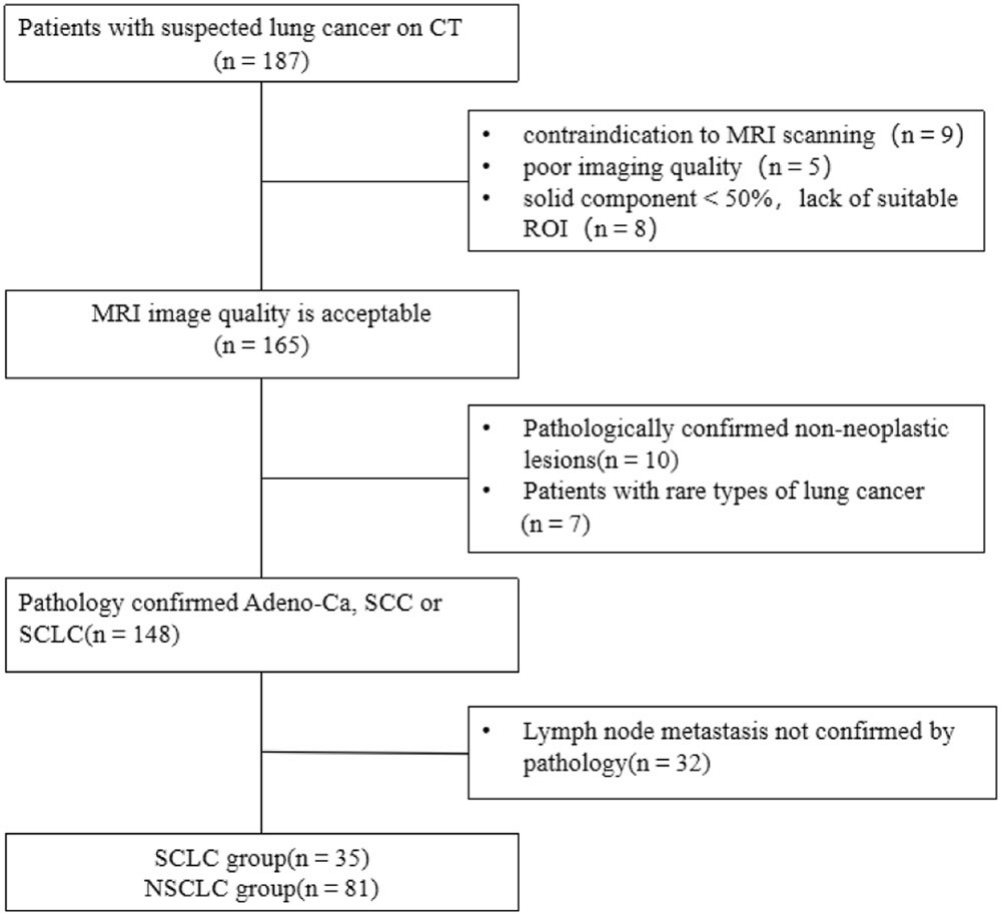
Flowchart of patient selection. Adeno-Ca, adenocarcinoma; CT, computed tomography; MRI, magnetic resonance imaging; SCC, squamous cell carcinoma; NSCLC, non-small-cell lung cancer; ROI, region of interest; SCLC, small-cell-lung cancer

This prospective study was approved by the local institutional review board. All participants provided written informed consent. Between September 2021 and October 2023, 187 individuals were enrolled prospectively. The inclusion criteria were: (1) individuals exhibited computed tomography (CT) findings indicative of lung cancer, (2) no treatment was initiated, and (3) the longest diameters of the pulmonary tumor were measured on CT ≥ 1.5 cm. The exclusion criteria were: (1) calcification or necrosis was more than one-third, precluding the identification of a suitable region of interest (ROI) > 50 mm^2^, and (2) poor imaging quality. Within 1 week after the MRI scan, all patients were diagnosed by histological evaluation of surgical or biopsy specimens. Figure 1 shows the flowchart of patient selection.

### MRI acquisition

MRI was performed with a 3.0-T clinical magnetic resonance scanner (MAGNETOM Prisma; Siemens, Erlangen, Germany) equipped with a dedicated 18-channel body-phased array coil. Before the examination, respiratory training is implemented to boost patient cooperation and mitigate motion artifacts induced by breathing. T2-weighted half-Fourier single-shot turbo spin-echo (HASTE) and BLADE sequences (Siemens, Erlangen, Germany) were performed first for lesion localization. The ZOOMit IVIM scan (*b*-values: 0 s/mm^2^, 20 s/mm^2^, 50 s/mm^2^, 100 s/mm^2^, 150 s/mm^2^, 200 s/mm^2^, 400 s/mm^2^, and 800 s/mm^2^) was then performed. To reduce the impact of magnetic field inhomogeneity, B1 field correction was conducted. Then followed by a T1 mapping scan using a variable flip angle three-dimensional (3D) volumetric interpolated breath-hold examination (VIBE) sequence. The flip angles were 3° and 15°. Subsequently, gadopentetate dimeglumine was injected at a dose of 0.1 mmol/kg, followed by a postcontrast T1 mapping sequence after 10 min. Detailed MRI protocols are shown in Table 1.

**Table 1:** Detailed MRI protocols

Sequence	Repetition time, (ms)	Echo time, (ms)	Section thickness, (mm)	Layer spacing, (mm)	Field of view, (mm^2^)	Scanning time, (s)	Respiratory compensation
T2WI-HASTE	1200	96	3.5	1.0	400 × 400	36	Hold breath
Fat-saturated T2WI-BLADE	2890	94	4.0	0.8	380 × 380	68	Hold breath
ZOOMit IVIM	3200	64	3.5	0.4	150 × 89.2	299	Breathe freely
T1 mapping	5.01	2.3	2.5	0.8	380 × 265	14	Hold breath

*BLADE* a vendor-specific implementation of the periodically rotated overlapping parallel lines with enhanced reconstruction (PROPELLER) technique, *MRI* magnetic resonance imaging, *HASTE* half-Fourier single-shot turbo spin-echo, *ZOOMit IVIM* ZOOM imaging technology intravoxel incoherent motion

### MRI evaluation and data processing

All the collected data were transferred to the workstation (syngo. via, VB20A, Siemens Healthcare, Erlangen, Germany). In the blinded to clinical and histopathological information (excluding lung cancer diagnosis), two experienced radiologists (H.Z.H. and P.Z., with experience of 10 years and 15 years in pulmonary tumor imaging, respectively) independently analyzed all scans to compare reproducibility in measurements between observers. The average values obtained by two radiologists were utilized for subsequent statistical analysis.

All the images were post-processed using prototypic software (MR Body Diffusion Toolbox; Siemens Healthcare, Erlangen, Germany) to extract the ADC, *D*, pseudo-diffusion coefficient (D^*^), and perfusion fraction (*f* ) values from the ZOOMit IVIM dataset. Manually delineated ROIs on the largest cross-section of the tumor and the two adjacent layers above and below, avoiding necrosis and hemorrhage. Each patient’s CT and MRI images (acquired by T2-weighted HASTE and fat-saturated BLADE) served as the reference during the ROIs outlining. ROIs were drawn on the postcontrast T1 maps to obtain the T1 values after contrast administration (T1_post_) and maintain consistency with the ROIs on the IVIM maps. The ROIs were then copied onto the precontrast T1 maps (T1_pre_). In addition, the T1 values of the blood pool were measured from the arteries at the same level of the lesion (T1_bloodpre_ and T1_bloodpost_, respectively). The hematocrit of each patient was obtained within 1 week after the MRI scans. The calculation of ECV is based on the following formula:

$${{{{{\rm{ECV}}}}}}= 	 \, (1-{{{{{\rm{hematocrit}}}}}})\times [(1/{{{{{\rm{T}}}}}}{1}_{{{{{{\rm{post}}}}}}}-1/{{{{{\rm{T}}}}}}{1}_{{{{{{\rm{pre}}}}}}})\\ 	 /(1/{{{{{\rm{T}}}}}}{1}_{{{{{{\rm{bloodpost}}}}}}}-1/{{{{{\rm{T}}}}}}{1}_{{{{{{\rm{bloodpre}}}}}}})]$$

### Statistical analysis

SPSS (version 26.0, IBM Corp., USA) and MedCalc software (version 15.2.2, Mariakerke, Belgium) were used for statistical analysis. The Shapiro–Wilk test was employed to assess the normality of the data. Continuous variables were presented as mean ± standard deviation. The inter-observer agreement of various parameters was evaluated using the intraclass correlation coefficient (ICC), with the average of measurements across three levels considered as the final result for each doctor’s measurement. Student’s *t*-test (for normally distributed data) or Mann–Whitney *U*-test (for non-normally distributed data) were utilized to analyze differences between parameters in NSCLC and SCLC, SCC and Adeno-Ca, and between cases without and with LNM. The receiver operating characteristic (ROC) curve analysis was conducted on parameters that exhibited statistical significance between groups, and the area under the curve (AUC) was determined. Optimal cutoff values for predicting SCLC, SCC, Adeno-Ca, and LNM status were defined when the Youden index reached its maximum value, and diagnostic performance was evaluated based on these cutoff values. The AUCs of various parameters were compared using the DeLong test. A *p*-value < 0.05 indicated statistical significance.

## Results

**Fig. 2:**
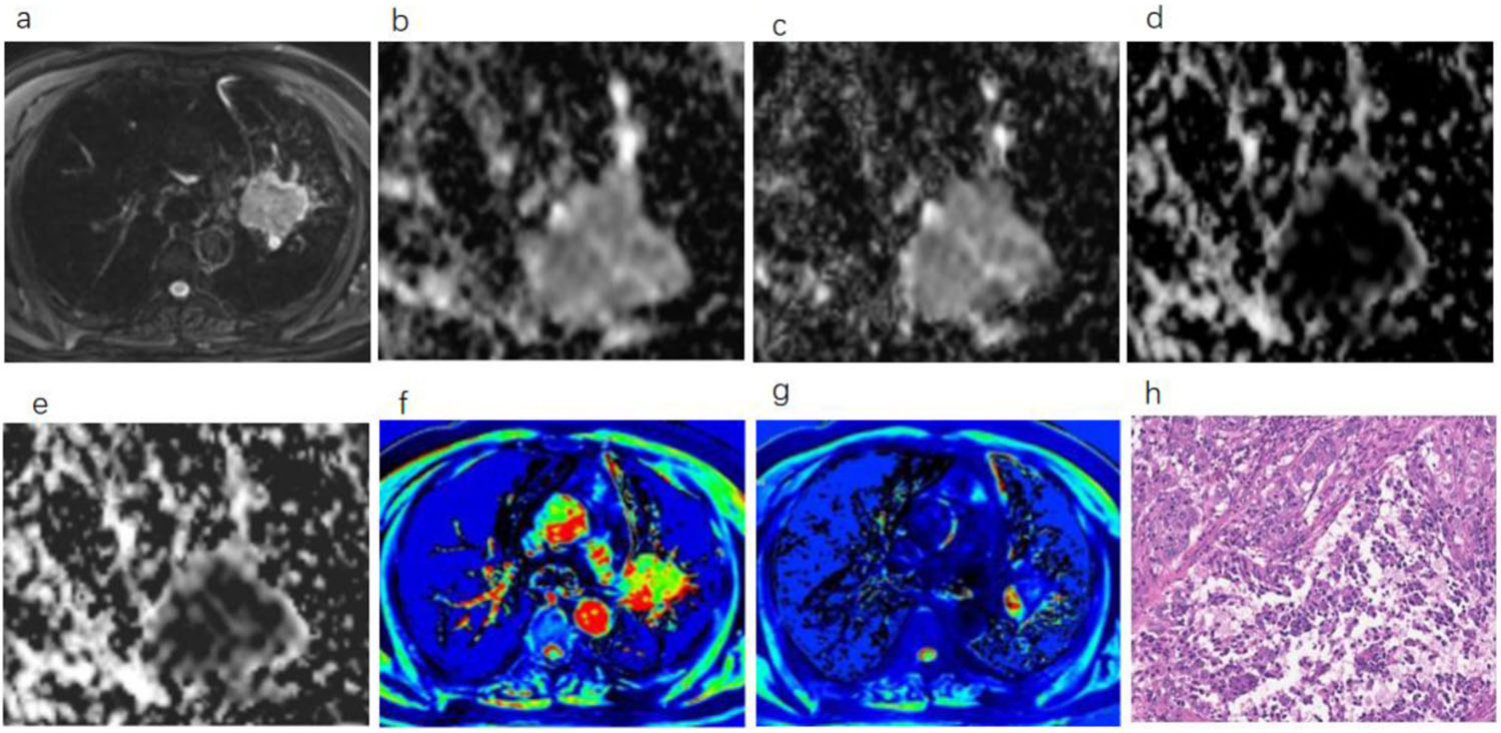
A 58-year-old male patient with SCC. **a** Axial image of T2-BLADE; **b**–**e** IVIM parameters ADC, *D*, D^*^, and *f* maps; **f** native T1 pseudo-color map; **g** postcontrast T1 pseudo-color map; and (**h**) pathologic picture (HE × 10). ADC, apparent diffusion coefficient; *D*, true diffusion; D^*^, pseudo-diffusion; *f*, perfusion fraction; HE, hematoxylin and eosin; IVIM, intravoxel incoherent motion

A total of 116 patients with lung cancer were enrolled (26 females, 90 males; mean age 61.00 ± 8.30 years, range 39–77 years). Among them were 81 cases with NSCLC (47 patients with Adeno-Ca and 34 patients with SCC; 39 patients without LNM and 42 patients with LNM) and 35 cases with SCLC. The determination of pathological subtypes and LNM status relies on pathological examination. The patients were categorized into the Adeno-Ca, SCC, and SCLC groups. Patients with NSCLC were further subdivided into the LNM (+) and LNM (−) groups based on their lymph node status. Table 2 summarizes the clinicopathologic characteristics of the patients. Representative cases are shown in Fig. 2.

**Table 2:** Clinicopathologic characteristics of patients included in this study

	Adeno-Ca, (*N* = 47)	SCC, (*N* = 34)	SCLC, (*N* = 35)
Age at diagnosis (year), mean ± SD	60.79 ± 8.19	60.97 ± 8.03	61.31 ± 8.91
Sex			
Male	32	31	27
Female	15	3	8
LNM			
Present	23	19	31
Absent	24	15	4
Lesion location			
RUL	18	12	19
RML	2	1	2
RLL	6	2	4
LUL	11	12	6
LLL	10	7	4
Lesion size (cm), mean ± SD	3.4 ± 2.44	3.4 ± 1.31	3.81 ± 1.47

*Adeno-Ca* adenocarcinoma, *LLL* left lower lobe, *LUL* left upper lobe, *RLL* right lower lobe, *RML* right middle lobe, *RUL* right upper lobe, *SCC* squamous cell carcinoma, *SCLC* small-cell lung cancer, *SD* standard deviation

### Inter-observer variability for IVIM parameters and ECV

The overall inter-observer consistency was good to excellent, with an ICC range of 0.713–0.898 (Table 3).

**Table 3:** Inter-observer variability for MRI parameters

Parameters	ICC	95% CI
ADC	0.872	0.816–0.912
*D*	0.898	0.853–0.929
D^*^	0.713	0.586–0.802
*f*	0.879	0.825–0.916
ECV	0.896	0.850–0.928

*ADC* apparent diffusion coefficient, *CI* confidence interval, *D* true diffusion coefficient, D^*^ pseudo-diffusion coefficient, *ECV* extracellular volume fraction, *f* perfusion fraction, *ICC* intraclass correlation coefficient, *MRI* magnetic resonance imaging

### Comparison of IVIM parameters and ECV between NSCLC and SCLC

The ADC, D, and ECV values in SCLC were considerably lower compared with NSCLC (all *p* < 0.001, Table 4). However, there was no statistically significant difference in the f and D^*^ values between the SCLC and NSCLC groups.

**Table 4:** Comparison of IVIM parameters and ECV among various pathologic subtypes of lung cancer and in NSCLC with and without LNM

Parameter	SCLC	NSCLC	*p*	NSCLC					
				Adeno-Ca	SCC	*p*	LNM (−)	LNM (+)	*p*
ADC (× 10^−3^ mm^2^/s)	0.920 ± 0.130	1.172 ± 0.111	< 0.001	1.179 ± 0.120	1.143 ± 0.107	0.164	1.164 ± 0.123	1.164 ± 0.110	0.982
*D* (× 10^−3^ mm^2^/s)	0.869 ± 0.095	1.053 ± 0.091	< 0.001	1.084 ± 0.092	1.010 ± 0.071	< 0.001	1.033 ± 0.087	1.071 ± 0.093	0.062
D^*^ (× 10^−3^ mm^2^/s)	14.785 ± 5.221	15.123 ± 4.655	0.730	15.235 ± 4.782	14.970 ± 4.540	0.802	14.956 ± 4.724	15.279 ± 4.641	0.757
*f* (%)	24.862 ± 8.209	24.731 ± 5.075	0.931	24.871 ± 4.879	24.537 ± 5.443	0.772	22.896 ± 5.136	26.435 ± 4.431	0.001
ECV	0.200 ± 0.049	0.303 ± 0.070	< 0.001	0.297 ± 0.070	0.310 ± 0.070	0.423	0.258 ± 0.055	0.344 ± 0.054	< 0.001

*ADC* apparent diffusion coefficient, *Adeno-Ca* adenocarcinoma, *D* true diffusion coefficient, D^*^ pseudo-diffusion coefficient, *ECV* extracellular volume fraction, *f* perfusion fraction, *IVIM* intravoxel incoherent motion, *LNM* lymph node metastasis, *NSCLC* non-small-cell lung cancer, *SCC* squamous cell carcinoma, *SCLC* small-cell lung cancer

### Comparison of IVIM parameters and ECV between SCC and Adeno-Ca

The *D* value in Adeno-Ca was substantially higher compared with SCC (*p* < 0.001, Table 4). However, there was no statistically significant difference in other IVIM parameters and ECV between Adeno-Ca and SCC groups (all *p* > 0.05, Table 4).

### Comparison of IVIM parameters and ECV between NSCLC without and with LNM

The *f* and ECV values in the LNM (+) group were markedly higher compared with the LNM (−) group (*p* < 0.05, *p* < 0.001, Table 4). However, there was no substantial difference in the ADC, *D*, and D^*^ values between NSCLC with LNM (+) and LNM (−) groups.

### IVIM parameters and ECV in distinguishing between NSCLC and SCLC, as well as SCC and Adeno-Ca, and predicting the LNM status in patients with NSCLC

**Fig. 3:**
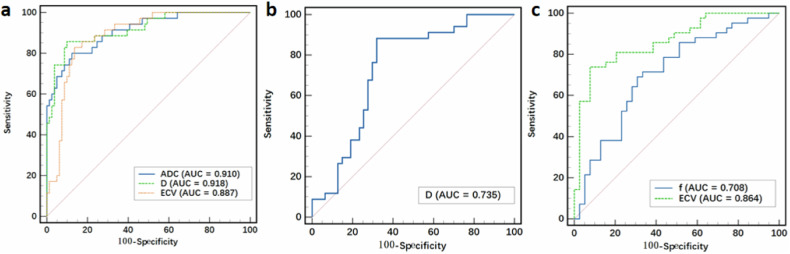
ROC curves of IVIM parameters and ECV for differentiating SCLC from NSCLC (**a**), Adeno-Ca from SCC (**b**), and NSCLC with LNM from NSCLC without LNM (**c**). Adeno-Ca, adenocarcinoma; AUC, area under the ROC curve; ECV, extracellular volume fraction; IVIM, intravoxel incoherent motion; LNM, lymph node metastasis; NSCLC, non-small-cell lung cancer; ROC, receiver-operating characteristic; SCC, squamous cell carcinoma; SCLC, small-cell lung cancer

ROC analysis showed that ADC, *D*, and ECV values had high diagnostic performance in distinguishing between NSCLC and SCLC, with the D value having the largest AUC of 0.918 (Table 5 and Fig. 3). However, there was no significant disparity in diagnostic performance among the ADC, D, and ECV values (DeLong test: all *p* > 0.05, *Z*: 0.449–0.757). The *D* value demonstrated satisfactory diagnostic value in distinguishing Adeno-Ca from SCC, with an AUC of 0.735 (Table 5 and Fig. 3). In predicting LNM status in NSCLC, the ECV showed higher diagnostic value compared with the *f* value (DeLong test: *p* < 0.05, *Z*: 2.190) (Table 5 and Fig. 3).

**Table 5:** Diagnostic performance of IVIM parameters and ECV for differentiating lung cancer subtypes and LNM status in NSCLC

Parameter	AUC	Cutoff	Sensitivity, (%)	Specificity, (%)	Youden index
SCLC vs NSCLC					
ADC	0.910 (0.843–0.955)	0.991 × 10^−3^ mm^2^/s	80.0	87.7	0.677
* D*	0.918 (0.853–0.961)	0.945 × 10^−3^ mm^2^/s	85.7	90.1	0.758
ECV	0.887 (0.815–0.939)	0.231	82.9	86.4	0.693
Adeno-Ca vs SCC					
* D*	0.735 (0.625–0.827)	1.077 × 10^−3^ mm^2^/s	88.2	68.1	0.563
NSCLC with vs without LNM					
* f*	0.708 (0.597–0.804)	25.061%	69	69.2	0.383
ECV	0.864 (0.770–0.930)	0.319	73.8	92.3	0.661

*ADC* apparent diffusion coefficient, *Adeno-Ca* adenocarcinoma, *AUC* area under the receiver-operating characteristic curve, *D* true diffusion coefficient, *ECV* extracellular volume fraction, *f* perfusion fraction, *IVIM* intravoxel incoherent motion, *LNM* lymph node metastasis, *MRI* magnetic resonance imaging, *NSCLC* non-small-cell lung cancer, *SCC* squamous cell carcinoma, *SCLC* small-cell lung cancer

## Discussion

This study indicated that the ADC, *D*, and ECV values effectively distinguished SCLC from NSCLC. Additionally, the *D* value demonstrated its utility in discerning Adeno-Ca from SCC. The ECV and *f* values demonstrated significance in predicting the LNM status in NSCLC, with the ECV exhibiting higher diagnostic accuracy. Therefore, IVIM and ECV can serve as non-invasive biomarkers for assisting in the pathological classification and LNM status assessment of lung cancer patients, providing valuable information for clinical decision-making.

Various subtypes of lung cancer have distinct biological behaviors and treatment strategies. Early diagnosis is vital for developing appropriate treatment plans and assessing prognosis. Our study demonstrated that the ADC, *D*, and ECV values in the SCLC group were considerably lower compared with the NSCLC group, and the *D* value in the SCC group was substantially lower compared with the Adeno-Ca group, which corresponded with the results reported by Fang et al [13] and Zheng et al [8]. The ADC, *D*, and ECV values collectively reflect the cellular density and extracellular space within the tissue [8, 9]. SCLC is highly cellular, with large nuclei, scant cytoplasm, and little extracellular space, restricting the diffusion motion of water [8]. Hence, the ADC, *D*, and ECV values in the SCLC group are substantially lower compared with the NSCLC group. SCC has a denser cellular structure compared with Adeno-Ca [14], but no substantial difference in the ADC and ECV values was observed between the two subtypes. A recent meta-analysis reinforced that the ADC value alone might not effectively distinguish NSCLC subtypes [15]. This indicates that the *D* value, better representing the diffusion motion of water molecules independent of tissue perfusion effects, surpasses the ADC value in this aspect. Few studies have reported on ECV in lung cancer, necessitating further studies to confirm potential differences between the subtypes.

Both D^*^ and *f* are perfusion-related parameters. Our findings suggest no remarkable differences in D^*^ and *f* values between various pathologic types of lung cancer. Considerable controversy still exists regarding the values of *f* and D^*^ in lung diseases. Wang et al [16] and Deng et al [17] observed that the *f* value could differentiate inflammatory lesions from lung cancer. Liu et al [18] suggested its value in assessing the pathologic grade evaluation of Adeno-Ca. However, numerous literature sources have argued against using D^*^ and *f* values as reliable indicators for differentiating malignant and benign lung tumors and their pathologic types. On the one hand, microcirculation perfusion of lung tumors of various subtypes is similar to a certain extent [19]. On the other hand, D^*^ is sensitive to the signal-to-noise ratio and has poor reproducibility [8], which explains the low ICC value of D^*^ in our study. While there is an ongoing debate on the significance of *f* and D^*^ values in lung disease, they have important potential for reflecting information about lesion perfusion. It needs to be further explored by standardizing the scanning parameters and expanding the sample size.

LNM is a key factor affecting the clinical stage. Precise preoperative assessment of the LNM status is of utmost importance for formulating treatment plans and evaluating prognosis. In our study, the LNM (+) group exhibited elevated ECV and f values compared with the LNM (−) group. This observation is consistent with the findings in cervical cancer [12, 20], where higher *f* and ECV values in cervical cancers were associated with increased invasiveness and a greater likelihood of pelvic LNM or lymphovascular space invasion. A higher *f* value in the metastatic group indicates an elevated microcirculation proportion, increased neovascularization, active cell proliferation, and enhanced invasiveness [21]. One common approach of LNM is through invasion of the microvascular system [22]. Therefore, NSCLC with higher blood perfusion is more prone to LNM. Cancer-associated fibroblasts in tumor tissue contribute to extracellular matrix stiffening, leading to an elevation in interstitial fluid pressure and enlargement of the extracellular compartment [23]. These factors can promote the penetration of shed tumor cells into lymphatic channels and microvasculature [24]. The rise in interstitial fluid pressure and the enlargement of the extracellular interstice led to an increase in ECV, thus heightening the probability of LNM.

Moreover, we assessed the abilities of ECV and *f* values in predicting the LNM status of NSCLC. ECV demonstrated superior diagnostic performance. ECV, expressed as a percentage and derived from the ratio of T1 values, exhibits less susceptibility to variations in field strengths, vendors, and acquisition techniques. Recent studies have indicated that T1 values exhibit strong scan consistency and repeatability in patients with lung cancer [25]. In our study, the T1 mapping acquired by the 3D T1-weighted VIBE sequence necessitates only 14 s to complete a single examination, allowing its execution within a single breath-hold, even for patients with concurrent emphysema or chronic obstructive pulmonary disease. Using ECV to predict LNM status in NSCLC may offer benefits to LNM (+) patients through preoperative intervention or postoperative anti-tumor treatment. Wang et al demonstrated that ECV can predict lymphatic vessel space invasion in cervical cancer patients without LNM, which is of clinical significance for the selection of surgical methods and prognosis assessment [12]. Our study extends research on ECV in the realm of lung cancer, offering fresh insights and potential avenues for personalized diagnosis and treatment across breast cancer, colorectal cancer, and other diseases.

This study had certain limitations. First, the sample size was small and obtained from a single center. Second, the quantity and distribution of *b*-values in IVIM lack uniform standards. Increasing the number of *b*-values enhances the image quality but also leads to a longer acquisition time. In this study, *b*-values were selected based on previous research [18]. Third, our study focused on lesions with a diameter larger than 1.5 cm, as smaller lesions are prone to interference from large cardiac vessels and respiratory motion, resulting in poor signal-to-noise ratios and suboptimal image quality [16]. However, as MRI technology advances, future research will include lesions smaller than 1.5 cm.

## Conclusion

In summary, IVIM parameters and ECV are promising tools for distinguishing lung cancer subtypes and predicting LNM in NSCLC. They offer clinicians a quantitative tool to promptly determine patient pathology types and identify high-risk cases of LNM, thereby aiding in the formulation of personalized treatment strategies.

## Data Availability

The data used or analyzed during the current study are available from the corresponding author on reasonable request.

## Abbreviations

ADC: Apparent diffusion coefficient
Adeno-Ca: Adenocarcinoma
AUC: Area under the curve
CT: Computed tomography
*D*: True diffusion coefficient
*D*^*^: Pseudo-diffusion coefficient
3D: Three-dimensional
ECV: Extracellular volume fraction
*f*: Perfusion fraction
ICC: Interclass correlation coefficient
IVIM: Intravoxel incoherent motion
LNM: Lymph node metastasis
MRI: Magnetic resonance imaging
NSCLC: Non-small-cell lung cancer
ROC: Receiver operating characteristic
ROI: Region of interest
SCC: Squamous cell carcinoma
SCLC: Small-cell lung cancer
VIBE: Volumetric interpolated breath-hold examination
